# Lived Experiences, Social Support Systems, and Coping Mechanisms Among Parenting Adolescents With Depression in Southwestern Uganda: A Qualitative Phenomenological Study

**DOI:** 10.7759/cureus.112824

**Published:** 2026-07-16

**Authors:** Scovia Atwiine, Viola N Nyakato, Daniel Atwine, Elizabeth Kemigisha

**Affiliations:** 1 Community Health, Mbarara University of Science and Technology, Mbarara, UGA; 2 Department of Clinical Research, SRF Research and Training Centre, Mbarara, UGA; 3 Health and Wellbeing, African Population and Health Research Center, Nairobi, KEN

**Keywords:** adolescent parenthood, coping mechanisms, depression, mental health, qualitative research, social support, southwestern uganda

## Abstract

Background: Parenting during adolescence is associated with significant emotional, social, and economic challenges that increase vulnerability to depression and psychological distress. In sub-Saharan Africa, adolescent parents frequently experience stigma, school dropout, poverty, weak social support systems, and limited access to adolescent-friendly mental health services. However, limited qualitative evidence exists regarding the lived experiences of adolescent mothers and fathers with depression in Uganda. This study explored the lived experiences, social support systems, and coping mechanisms of parenting adolescents with depression in southwestern Uganda.

Methods: A qualitative phenomenological study was conducted between March 2025 and January 2026 in selected health facilities and neighboring communities in Mbarara District, southwestern Uganda. Purposive sampling was used to recruit adolescent mothers and fathers aged 10-19 years with depressive symptoms, as well as key informants, including caregivers, community leaders, Village Health Team (VHT) members, and health workers. Data were collected through in-depth interviews and key informant interviews using semistructured interview guides. Interviews were audio-recorded, transcribed verbatim, translated into English, and thematically analyzed using both deductive and inductive coding approaches supported by NVivo version 12.2 (QSR International Pty Ltd., Melbourne, VIC, Australia). Bronfenbrenner's Ecological Systems Theory guided the interpretation of the findings.

Results: A total of 33 participants were interviewed, including 12 (36.3%) adolescent mothers, six (18.2%) adolescent fathers, and 15 (45.5%) key informants. Three major thematic domains emerged: lived experiences, social support systems, and coping mechanisms. Parenting adolescents described significant emotional and psychological distress characterized by persistent worry, hopelessness, social isolation, and severe emotional pain. Depression was strongly linked to poverty, school dropout, unemployment, partner abandonment, stigma, and family rejection. Social support was obtained primarily from parents, grandparents, partners, health workers, and community leaders, although these support systems were inconsistent and often limited. Adolescents primarily relied on informal coping mechanisms, including prayer, peer support, casual labor, resilience, and acceptance of parenthood.

Conclusion: Depression among parenting adolescents in southwestern Uganda is shaped by complex interactions among emotional distress, socioeconomic hardship, stigma, and limited psychosocial support systems. Strengthening adolescent-friendly mental health services, family support systems, community sensitization, educational reintegration, and economic empowerment programs may improve the psychosocial well-being of parenting adolescents.

## Introduction

Adolescent pregnancy and parenting remain major public health and social challenges in sub-Saharan Africa, with important consequences for mental health, education, family relationships, and long-term socioeconomic well-being. Adolescence is already a period of major biological, psychological, and social transition; when pregnancy and parenting occur during this stage, young people are often forced to assume adult responsibilities before they are emotionally, socially, or economically prepared [1,2]. In many African settings, this transition is further complicated by poverty, school dropout, partner abandonment, family conflict, stigma, and limited access to adolescent-friendly health and psychosocial services.

Depression is one of the most frequently documented mental health problems among pregnant and parenting adolescents. Global evidence suggests that depressive symptoms affect a substantial proportion of adolescents, with the study by [3] reporting a high pooled prevalence of elevated depressive symptoms among adolescents. African studies similarly show a considerable burden, with depressive symptoms reported among pregnant and parenting adolescents in Burkina Faso, Malawi, Kenya, and Rwanda [4-6]. Beyond depression, parenting adolescents may experience anxiety, stress, post-traumatic symptoms, emotional distress, and suicidal thoughts, especially where pregnancy is unintended, socially condemned, or unsupported [7,8].

The mental health burden among parenting adolescents is shaped by multiple and interacting factors. At the individual level, low educational attainment, unintended pregnancy, HIV infection, traumatic experiences, and low self-esteem have been associated with depressive symptoms [4,5,9]. At the family level, lack of parental support, paternity denial, intimate partner violence, and partner abandonment increase vulnerability to emotional distress [10,11]. At the community level, stigma, gossip, peer rejection, and unsafe neighborhoods further deepen social isolation and reduce access to support [12,13]. These multilevel influences are consistent with Bronfenbrenner’s Ecological Systems Theory, which recognizes that adolescent well-being is shaped by interactions across individual, interpersonal, community, institutional, and societal contexts [14].

In Uganda, adolescent pregnancy remains a major concern, particularly in rural and peri-urban settings where young mothers may face strong social judgment, expulsion from school, family rejection, and loss of future aspirations [8]. Although existing studies have documented depression and psychosocial vulnerability among adolescent mothers, fewer studies have explored the lived experiences of both adolescent mothers and fathers with depression, including how they access support and cope with early parenthood. This study, therefore, examined the lived experiences, social support systems, and coping mechanisms among adolescent mothers and fathers aged 10-19 years with depression in southwestern Uganda.

## Materials and methods

Study design and theoretical orientation

This study adopted a qualitative phenomenological design guided by an interpretivist paradigm to explore the lived experiences, social support systems, and coping mechanisms among adolescent mothers and fathers with depression in southwestern Uganda. A phenomenological approach was considered appropriate because it enables an in-depth exploration of how individuals perceive, interpret, and make meaning of their lived experiences and emotional realities [15]. The study specifically sought to understand how parenting adolescents experience depression within their social, family, and community contexts.

The analysis was conceptually informed by Bronfenbrenner’s Ecological Systems Theory, which recognizes that adolescent well-being is shaped by interactions across multiple environmental levels, including individual, interpersonal, community, institutional, and societal contexts [16]. This framework guided the interpretation of how family relationships, stigma, poverty, community support, and broader social structures influenced depressive symptoms and coping mechanisms among parenting adolescents.

Study setting

The study was conducted between March 2025 and January 2026 in selected health facilities and neighboring communities in Mbarara District, southwestern Uganda. Mbarara District was purposively selected because of its high burden of adolescent pregnancy and parenthood, as highlighted in district and national reports [17,18].

Data were collected from Rubindi Health Centre III, Rubaya Health Centre III, and Bwizibwera Health Centre IV, which had reported high numbers of adolescent antenatal and postnatal visits. Additional participants were recruited from surrounding communities to ensure the inclusion of adolescents who were not actively utilizing healthcare services.

Study population and eligibility criteria

**Figure 1:**
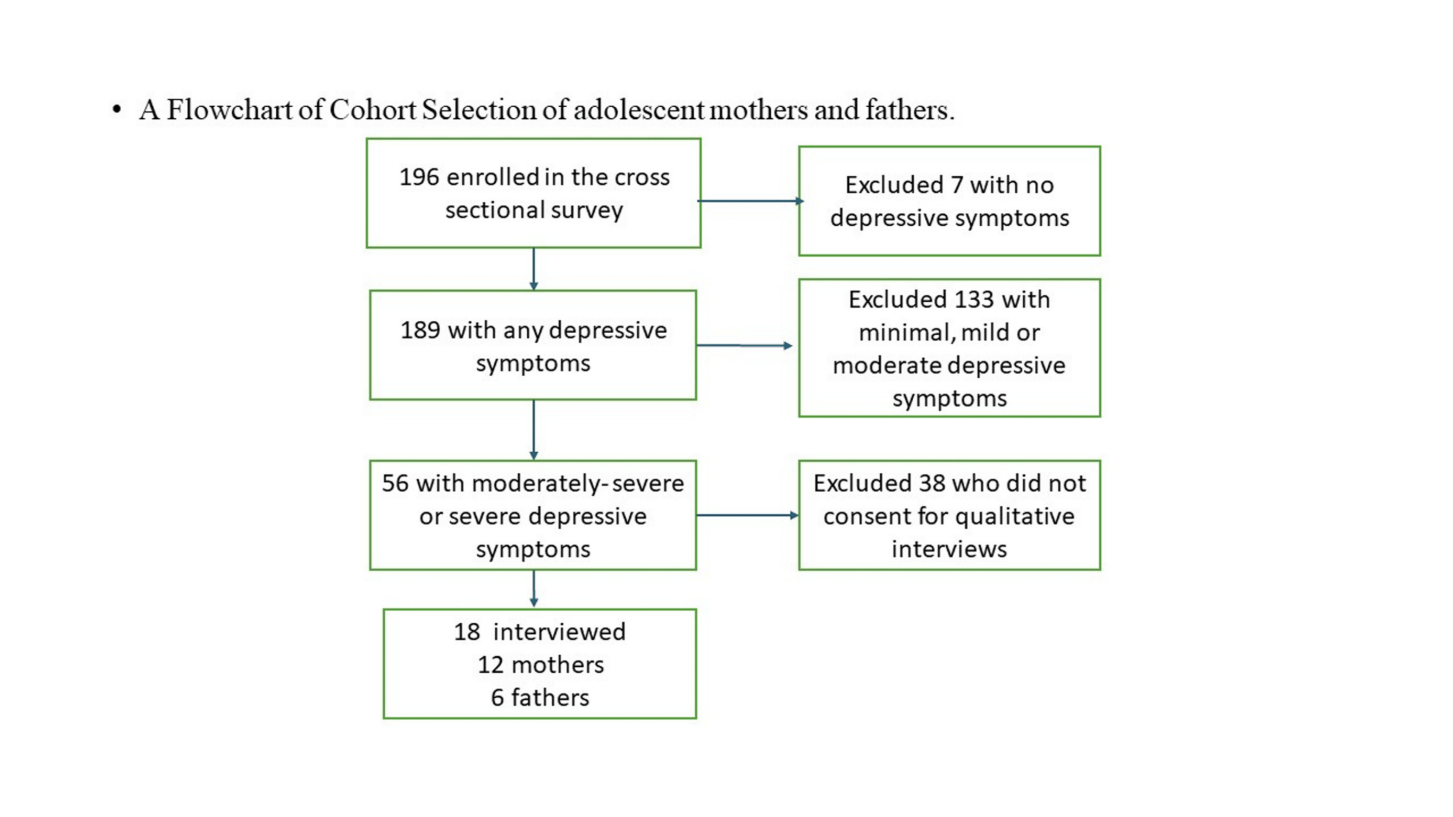
Cohort selection of adolescent mothers and fathers

The study population comprised adolescent mothers and fathers aged 10-19 years who had experienced depressive symptoms and were residing within the study area. The study sample was drawn from the quantitative study that was conducted to establish the prevalence of depressive symptoms among adolescent mothers and fathers in southwestern Uganda. Depressive symptoms were assessed using the Patient Health Questionnaire-9 (PHQ-9), which classifies depression severity as mild (5-9), moderate (10-14), moderately severe (15-19), and severe (20-27). Participants with a PHQ-9 score of ≥10 were purposively selected, as this cut-off is recommended for identifying probable depression with good diagnostic accuracy [19]. Therefore, adolescent mothers and fathers who presented with PHQ-9 scores of ≥10 were enrolled in the qualitative study to explore their lived experiences and social support systems, as presented in Figure 1.

The study additionally included key informants who interacted directly with parenting adolescents and were involved in providing support or services. These included parents or caregivers, health workers, Village Health Team (VHT) members, community leaders, including Local Council I chairpersons and Community Development Officers.

Parenting adolescents who provided written informed consent were eligible for participation. Adolescents who were unable to provide informed consent due to severe mental or cognitive impairment were excluded. Non-parenting adolescents, non-Ugandan nationals, refugees, and adult men outside the adolescent age category were also excluded from the study.

Sampling and recruitment procedures

Purposive sampling was used to recruit participants with relevant experiences and knowledge related to adolescent parenthood and depression. Parenting adolescents with depressive symptoms was identified with the support of health workers, VHT members, and adolescent peer leaders from health facilities and neighboring communities. This approach enabled recruitment of both facility-based and community-based participants, including adolescents who were not regularly accessing healthcare services.

Key informants were purposively selected based on their involvement in adolescent care, counseling, protection, or community support services. Recruitment continued until thematic saturation was achieved, defined as the point at which additional interviews no longer generated new themes or insights relevant to the study objectives.

Data collection procedures

Qualitative data were collected through in-depth interviews (IDIs) and key informant interviews (KIIs). IDIs were conducted among adolescent mothers and fathers with depressive symptoms to explore their emotional experiences, challenges associated with adolescent parenthood, coping strategies, and available support systems. KIIs were conducted with parents or caregivers, health workers, VHT members, and community leaders to obtain broader perspectives regarding community attitudes, support systems, and challenges affecting parenting adolescents. Parents were considered key informants (KIIs) and provided general information about the experiences of parenting adolescents in the community.

Semi-structured interview guides with open-ended questions were developed by the research team and used to facilitate discussion while allowing participants the flexibility to describe their experiences in their own words. Interviews were conducted in Runyankole, the predominant local language in the study area, by the principal investigator with support from two trained research assistants experienced in qualitative interviewing. All interviews were audio-recorded with participants' consent to ensure the completeness and accuracy of the data. Field notes were also maintained during interviews to capture contextual observations and nonverbal expressions.

Data quality assurance

Research assistants received training in qualitative interviewing techniques, ethical conduct, confidentiality, and the management of emotionally sensitive discussions before data collection. Interview guides were pretested to assess clarity, cultural appropriateness, and consistency with the study objectives.

Credibility of the findings was enhanced through triangulation of participant categories, inclusion of multiple stakeholder perspectives, prolonged engagement with transcripts, and the use of analytical memos throughout the analytical process. We used data source triangulation by collecting data from different stakeholder groups (adolescents, caregivers, local leaders, VHT members, and health workers) and method triangulation by combining IDIs and KIIs. Findings were compared across sources and methods to enhance the credibility of the results.

Data management and analysis

All audio-recorded interviews were transcribed verbatim and translated from Runyankole into English before analysis. Data collection and analysis occurred concurrently, allowing emerging findings to inform subsequent interviews and facilitate the exploration of developing themes. Participant data were deidentified before analysis, and all personal identifiers were removed to ensure confidentiality and protect participants' privacy.

Qualitative data were analyzed using thematic analysis guided by both deductive and inductive coding approaches. Deductive coding was informed by the study objectives, while inductive coding allowed additional contextual themes and meanings to emerge directly from participants' narratives [20]. Bronfenbrenner's Ecological Systems Theory guided the study [14].

NVivo version 12.2 (QSR International Pty Ltd., Melbourne, VIC, Australia) was used to facilitate the systematic organization and management of qualitative data. Interview transcripts were repeatedly reviewed to enhance familiarity with the data. Meaningful text segments were assigned descriptive codes, which were subsequently grouped into categories, subthemes, and overarching themes. Analytical memos were maintained throughout the coding process to document emerging interpretations, reflections, and relationships among themes.

The analysis generated major thematic domains related to the lived experiences, social support systems, and coping mechanisms of parenting adolescents with depression. Frequently occurring themes included emotional distress, stigma and discrimination, school dropout, poverty, financial hardship, family and partner support, spirituality, resilience, and acceptance of parenthood. Comparative analysis across participant groups further explored similarities and differences in perspectives among adolescent parents, caregivers, health workers, and community leaders.

Ethical considerations

Ethical approval was obtained from the Mbarara University of Science and Technology (MUST) Research Ethics Committee (Approval ID: MUST-2024-1387; April 2024) and the Uganda National Council for Science and Technology (UNCST) (Approval ID: SS3018ES; August 2024). Written informed consent was obtained from all participants before enrollment. Parenting adolescents were considered emancipated minors in accordance with UNCST guidelines. Participation was voluntary, and participants were informed of their right to decline participation or withdraw from the study at any time without consequences. Confidentiality, privacy, and anonymity were maintained throughout the study.

## Results

Respondent characteristics

A total of 33 participants were involved in the qualitative component of the study, comprising adolescent mothers, adolescent fathers, parents or caregivers, community leaders, health workers, and VHT members. Overall, female participants constituted the majority of respondents, largely because adolescent mothers represented the largest participant category in the study. Male respondents mainly consisted of adolescent fathers, community leaders, health workers, and one parent or caregiver. The diversity of participants across family, community, and health system levels strengthened the richness and credibility of the findings by allowing triangulation of perspectives regarding adolescent parenthood, depression, social support systems, and coping mechanisms.

The study included 12 (36.3%) adolescent mothers and six (18.2%) adolescent fathers, who formed the primary participant group for exploring the lived experiences of adolescent parenthood and depression. The inclusion of both adolescent mothers and fathers enabled the study to capture gendered experiences of emotional distress, coping, and social support related to early parenthood. In addition, four (12.1%) parents or caregivers, four (12.1%) community leaders, four (12.1%) health workers, and three (9.1%) VHT members participated as key informants, providing broader community and institutional perspectives regarding the psychosocial challenges faced by parenting adolescents. The participant characteristics are presented in Table 1.

**Table 1:** Respondents' characteristics IDIs, in-depth interviews; KIIs, key informant interviews; VHT, Village Health Team

Participant category	Male, n(%)	Female, n(%)	Total, n(%)	Data collection method
Adolescent mothers	0	12 (57.1)	12 (36.3)	IDIs
Adolescent fathers	6 (50)	0	6 (18.2)	IDIs
Parents/caregivers	1 (8.3)	3 (14.3)	4 (12.1)	KIIs
Community leaders	2 (16.7)	2 (9.5)	4 (12.1)	KIIs
Health workers	2 (16.7)	2 (9.5)	4 (12.1)	KIIs
VHT members	1 (8.3)	2 (9.5)	3 (9.1)	KIIs
Total	12	21	33	

Overall, female participants constituted the majority of respondents, largely because adolescent mothers represented the largest participant category in the study. Male respondents mainly consisted of adolescent fathers, community leaders, health workers, and one parent or caregiver. The diversity of participants across family, community, and health system levels strengthened the richness and credibility of the findings by allowing triangulation of perspectives regarding adolescent parenthood, depression, social support systems, and coping mechanisms.

The inclusion of participants from multiple stakeholder groups enabled triangulation of findings and provided a comprehensive understanding of the emotional, social, and economic realities surrounding adolescent parenthood in southwestern Uganda. Perspectives from adolescent parents were complemented by insights from caregivers, community leaders, VHT members, and health workers, thereby strengthening the trustworthiness and depth of the qualitative findings.

Lived experiences of adolescent mothers and fathers with depression

The findings showed that adolescent parenthood was experienced as a major emotional, social, and economic disruption. Adolescent mothers described shame, school dropout, abandonment, childcare burden, and uncertainty about the future. Adolescent fathers also experienced psychological pressure, particularly related to providing for a child without stable employment or adequate income.

Emotional distress was closely linked to poverty, stigma, family rejection, disrupted education, and weak partner support. Community judgment led some adolescents to withdraw from social life, while financial hardship intensified feelings of hopelessness. These findings suggest that depression among parenting adolescents is not only an individual mental health issue but also a response to social exclusion, economic insecurity, and limited support, as shown in Table 2.

**Table 2:** Lived experiences of adolescent mothers and fathers with depression IDIs, in-depth interviews; KIIs, key informant interviews; VHT, Village Health Team

Themes	Sub-themes	Key findings	Illustrative quotations	Respondent identification
Emotional and psychological distress	Persistent worry and overthinking	Adolescents experienced continuous worry about childcare, poverty, employment, and the future.	“I get a lot of thoughts and sometimes feel like I do not want to eat… because of thinking so much.”	Adolescent mother, IDI, March 2025
	Pressure to provide	Adolescent fathers reported stress from being expected to provide without stable work.	“Sometimes I keep thinking about how I will support the child when he starts school because I do not have a stable job.”	Adolescent father, IDI, March 2025
	Hopelessness and regret	Early parenthood disrupted education, work plans, and confidence in the future.	“I don’t think I will achieve my future plans… since the man deserted the family.”	Adolescent mother, IDI, March 2025
	Social isolation and self-stigma	Shame and fear of judgement led adolescents to withdraw from community life.	“Adolescents who become pregnant often begin to isolate themselves because they fear being laughed at in the community.”	Community leader, KII, Jan 2026
	Severe emotional distress	Some adolescents experienced overwhelming emotional pain due to abandonment, poverty, and lack of family support.	“I have a lot of thoughts because of pain… I am struggling alone because the man chased me away from his home.”	Adolescent mother, IDI, March 2025
Socio-economic challenges	School dropout	Pregnancy and parenting interrupted schooling for both adolescent mothers and fathers.	“When I became pregnant, I had to stop going to school because I was ashamed and also needed to take care of the baby.”	Adolescent mother, IDI, March 2025
	Poverty and hardship	Adolescents struggled to meet basic needs for themselves and their children.	“These young parents struggle because they do not have jobs or income, yet they must provide for the baby.”	Parent/Caregiver, IDI, Jan 2026
	Unemployment and dependency	Limited skills and lack of employment increased dependence on family or casual labor.	“If I don’t work per day, we can’t get what to eat… I have to work per day.”	Adolescent mother, IDI, March 2025
Social stigma and discrimination	Community gossip	Adolescents and their families were exposed to gossip, blame, and public judgment.	“People in the community start talking about the family and blaming the parents.”	Parent/Caregiver, IDI, Jan 2026
	Family rejection	Some adolescents were blamed, chased away, or denied support after pregnancy or fatherhood.	“They chased me out of the family… They blamed me of disappointing them.”	Adolescent father, IDI, Jan 2026
	Peer discrimination	Peers sometimes distanced themselves from adolescent parents.	“Fellow girls may distance themselves from someone who becomes pregnant because they fear being associated with her.”	VHT member, KII, Dec 2025
	Perceived failure	Communities often viewed adolescent parents as having spoiled their future.	“The community looks at these adolescents as failures because they started families when they had not yet developed themselves.”	Health worker, KII, Jan 2026

Social support systems available for adolescent mothers and fathers

Social support was central to how adolescent parents coped with early parenthood. Family support, especially from mothers and grandparents, helped adolescents manage childcare, food, household needs, and emotional stress. Where families were accepting and supportive, adolescents appeared better able to adjust to parenting responsibilities.

However, support was uneven. Some adolescent mothers were abandoned by partners, while some adolescent fathers lacked stable income despite wanting to provide. Community and institutional support was available through health workers, VHTs, community leaders, and non-governmental organizations, but these systems appeared limited in reach and consistency. The findings point to the need for stronger family-based, community-based, and facility-based support systems for parenting adolescents. Table 3 presents the social support systems available to adolescent mothers and fathers.

**Table 3:** Social support systems available to adolescent mothers and fathers NGO, non-governmental organizations, IDI, in-depth interview; KII, key informant interview

Themes	Sub-themes	Key findings	Illustrative quotations	Respondent identification
Family and partner support	Parental support	Parents provided childcare, emotional support, food, shelter, and financial assistance.	“My mother helps me take care of the baby when I have to do some work.”	Adolescent mother, IDI, March 2025
	Support to adolescent fathers	Some fathers received help from their own parents when unable to provide.	“When I fail to work and raise the money, they support by providing what is needed to my child.”	Adolescent father, IDI, March 2025
	Grandparent support	Grandparents played an important role in childcare and family support.	“Sometimes the grandmother takes care of the child when the young mother is busy.”	Parent/Caregiver, IDI, Dec 2025
	Partner involvement	Some adolescent fathers tried to support the mother and child despite limited resources.	“I try to help the mother of my child as much as I can… I work hard to ensure that I provide their needs.”	Adolescent father, IDI, March 2025
	Partner abandonment	Some adolescent mothers were abandoned after pregnancy, worsening distress and financial hardship.	“When he was called, he denied that he doesn’t know me and that he has never produced a child with me.”	Adolescent mother, IDI, March 2025
Community and institutional support	Health worker counseling	Health workers provided counselling, information, and emotional support.	“We try to counsel these young mothers and fathers so that they can cope with the situation and take good care of their children.”	Health worker, KII, Dec 2025
	Community leadership support	Community leaders encouraged families to support rather than reject adolescent parents.	“We encourage families to support these adolescents instead of rejecting them.”	Community leader, KII, Dec 2025
	NGO and group support	Community organizations supported counselling, saving groups, and poverty reduction efforts.	“We sit with them, counsel them and encourage them to save their money so that they can borrow and help themselves come out of poverty.”	Community leader, KII, Dec 2025

Coping mechanisms among adolescent mothers and fathers

Parenting adolescents used a combination of spiritual, emotional, social, and economic coping strategies. Prayer was a common source of comfort, while acceptance of parenthood helped some adolescents shift from regret toward responsibility. Peer support also helped adolescents feel less alone, especially when they interacted with others who understood their situation.

At the same time, many coping strategies were informal and survival-based. Some adolescents relied on casual labor to meet immediate needs, while others depended on family or friends for emotional support. The limited mention of structured psychosocial services suggests a gap in formal mental health support. Strengthening counseling, peer support groups, livelihood programs, and youth-friendly services could help adolescents move from survival coping toward supported recovery and resilience, as detailed in Table 4.

**Table 4:** Coping mechanisms among adolescent mothers and fathers IDI, in-depth interview; KII, key informant interview

Themes	Sub-themes	Key findings	Illustrative quotation	Respondent identification
Personal and spiritual coping	Prayer and spirituality	Prayer helped adolescents feel stronger, hopeful, and emotionally supported.	“I pray a lot because it helps me feel stronger and hopeful.”	Adolescent mother, IDI, March 2025
	Acceptance of parenthood	Some adolescents coped by accepting their new role and focusing on responsibility.	“I have accepted that I am now a parent, so I must work hard for my child and I.”	Adolescent father, IDI, 2025
Economic coping	Casual labor and self-reliance	Some adolescents worked in farms or informal jobs to survive and care for their children.	“I am trying to get better by working in people’s farms so that I can get money to start my own business.”	Adolescent mother, IDI, March 2025
Social coping	Peer support	Talking to friends with similar experiences reduced loneliness and emotional burden.	“Talking to friends who understand my situation helps me feel better.”	Adolescent mother, IDI, March 2025
Informal coping	Reliance on informal systems	Adolescents relied mainly on family, peers, prayer, and personal effort rather than structured mental health services.	“We try to counsel these young mothers and fathers so that they can cope with the situation.”	Health worker, KII, Dec 2025

## Discussion

This study explored how adolescent mothers and fathers with depression in southwestern Uganda experience early parenthood, the support systems available to them, and the coping mechanisms they use. The findings show that adolescent parenthood was not experienced as a single event but as a complex life disruption involving emotional distress, social stigma, economic hardship, interrupted education, unstable relationships, and limited formal social support. These findings reinforce existing evidence that depression among parenting adolescents is strongly shaped by social and structural conditions rather than individual vulnerability alone [11,21].

Emotional and psychological distress was a dominant finding. Adolescent mothers and fathers described persistent worry, hopelessness, regret, self-blame, and uncertainty about the future. For adolescent mothers, distress was often linked to pregnancy-related shame, school dropout, partner abandonment, and childcare responsibilities. For adolescent fathers, distress was strongly associated with pressure to provide despite unemployment and limited income. These findings are consistent with previous studies showing that parenting adolescents often experience depression, anxiety, emotional exhaustion, and psychological distress as they struggle to adjust to adult responsibilities at a young age [1,7]. Similar evidence from Kenya, Rwanda, Malawi, and Burkina Faso has shown that adolescent pregnancy and parenting are associated with high levels of depressive symptoms and psychosocial strain [5,6,21].

The study also found that socioeconomic hardship was central to the lived experiences of parenting adolescents. School dropout, poverty, unemployment, and financial dependency limited adolescents' ability to care for themselves and their children. These challenges contributed to emotional distress and reinforced feelings of hopelessness. This finding aligns with studies showing that adolescent pregnancy often initiates a cycle of social exclusion, educational disruption, and economic vulnerability [10,22]. The results also support previous evidence that poverty, food insecurity, low education, and limited employment opportunities are important determinants of poor mental health among young parents [23,24].

Stigma and discrimination were also prominent. Adolescents reported gossip, negative labeling, family rejection, peer discrimination, and being viewed as failures. This finding is consistent with literature showing that pregnancy-related stigma is a major psychosocial burden for adolescent mothers in Uganda and other African settings [8,13]. Importantly, this study also highlights stigma experienced by adolescent fathers, particularly when they were perceived as irresponsible or unable to provide. This suggests that interventions should not focus only on adolescent mothers but should also recognize the emotional and social experiences of adolescent fathers.

Family and partner support emerged as both protective and inconsistent. Adolescents who received support from parents, grandparents, or partners described better coping and greater emotional stability. However, others experienced partner denial, abandonment, or family rejection, which worsened distress. This finding is consistent with evidence that parental support, family cohesion, and positive partner relationships protect adolescent mental health, while a lack of support increases the risk of depression [25-27]. The findings highlight the importance of family-centered interventions that help parents and caregivers respond supportively rather than punitively to adolescent parenthood.

Community and institutional support systems were present but limited. Health workers, VHTs, community leaders, and local organizations provided counseling, advice, and encouragement, but these services were not consistently available or sufficiently structured. This reflects broader service gaps reported across sub-Saharan Africa, where pregnant and parenting adolescents often face limited adolescent-friendly mental health services, poor integration of mental health into maternal care, judgmental provider attitudes, and weak referral systems [7,11]. These findings point to the need to integrate mental health screening, counseling, and referral into routine antenatal, postnatal, and adolescent health services.

Coping strategies were mainly informal and survival-based. Adolescents relied on prayer, spirituality, peer support, acceptance of parenthood, casual labor, and personal resilience. These findings are consistent with studies from Uganda and Kenya showing that spirituality, peer networks, social comparison, acceptance, and support from friends can help young people manage psychological distress [25,28]. However, reliance on informal coping also reflects limited access to structured psychosocial care. While such strategies may offer emotional relief, they cannot substitute for accessible counseling, social protection, school reintegration, and livelihood support.

Overall, the findings support a socioecological understanding of depression among parenting adolescents. Emotional distress was shaped by individual experiences, family responses, community attitudes, economic hardship, and institutional service gaps. This means that effective responses must go beyond individual counseling. Programs should combine adolescent-friendly mental health services, family strengthening, community sensitization, peer support groups, school reentry support, economic empowerment, and protection from violence and stigma.

Strengths and limitations of the study

One of the major strengths of this study was the use of a qualitative phenomenological approach, which enabled an in-depth understanding of the lived realities of adolescent mothers and fathers with depression in southwestern Uganda. By including multiple participant groups, including adolescent parents, caregivers, community leaders, VHT members, and health workers, the study achieved triangulation of perspectives and generated a comprehensive understanding of the emotional, social, and economic contexts surrounding adolescent parenthood and depression. The inclusion of both adolescent mothers and fathers was particularly important because many previous studies have focused predominantly on adolescent mothers, with limited attention given to adolescent fathers and their psychosocial experiences.

The study also benefited from conducting interviews in both health facilities and surrounding communities, which allowed the inclusion of adolescents who were not actively utilizing formal healthcare services. This strengthened the diversity of experiences captured and improved the contextual relevance of the findings. In addition, the use of local-language interviews conducted by trained research assistants helped participants express sensitive experiences more freely and authentically.

However, several limitations should be considered when interpreting the findings. First, the study was conducted in selected facilities and communities in Mbarara District, which may limit the transferability of the findings to other settings with different social, cultural, or economic contexts. Second, because the study relied on self-reported experiences, some participants may have withheld sensitive information because of fear of stigma, shame, or emotional discomfort despite assurances of confidentiality. The cross-sectional nature of data collection also limited the exploration of how depressive experiences and coping mechanisms evolve over time.

Furthermore, although adolescents with depressive symptoms were purposively recruited, the study did not use a formal psychiatric diagnostic interview to confirm depression clinically. The findings, therefore, reflect participants' lived experiences of depressive symptoms and emotional distress rather than medically diagnosed depressive disorders. Nonetheless, the study provides important contextual insights into the psychosocial realities of parenting adolescents in resource-limited settings.

Implications for policy, practice, and programming

The findings of this study have important implications for adolescent mental health policy, maternal and child health programming, and community-based psychosocial support interventions. First, the study demonstrates that depression among parenting adolescents is closely linked to poverty, stigma, disrupted education, weak social support systems, and economic dependency. This suggests that mental health interventions targeting adolescent parents should not focus solely on clinical treatment but should adopt a broader socioecological and multisectoral approach.

At the health system level, there is a need to integrate routine mental health screening, counseling, and psychosocial assessment into antenatal, postnatal, and adolescent reproductive health services. Health workers providing maternal and adolescent health services should receive training in adolescent-friendly mental health care, including early identification of depressive symptoms, suicide risk assessment, trauma-informed counseling, and referral pathways. Community health workers and VHTs could also play an important role in identifying vulnerable adolescent parents and linking them to appropriate services.

The findings further highlight the need for family-centered and community-based interventions aimed at reducing stigma and strengthening support for adolescent parents. Community sensitization programs involving parents, religious leaders, schools, local leaders, and men could help challenge harmful attitudes that isolate or shame parenting adolescents. Family counseling and parenting support interventions may help families provide more supportive responses to adolescent pregnancy and parenthood.

Educational reintegration and economic empowerment programs are also critical. Many adolescents described school dropout, unemployment, and financial hardship as major drivers of emotional distress. Policies and programs that support adolescent mothers and fathers in returning to school, accessing vocational training, joining savings groups, or engaging in income-generating activities may improve both mental well-being and long-term socioeconomic outcomes.

In addition, the findings suggest an urgent need to strengthen adolescent-focused psychosocial support systems. Peer support groups, youth clubs, mentorship programs, and community safe spaces could provide adolescents with opportunities to share experiences, reduce isolation, and build resilience. Given the importance of spirituality identified in this study, faith-based institutions may also provide valuable platforms for psychosocial support and community reintegration when appropriately engaged.

Recommendations for future research

Future studies should explore the long-term mental health trajectories of parenting adolescents using longitudinal designs to better understand how depressive symptoms evolve across pregnancy, childbirth, and later parenting stages. Quantitative studies are also needed to determine the prevalence and predictors of depression among parenting adolescents in different Ugandan settings using validated screening and diagnostic tools.

Intervention-based research remains critically needed. There is limited evidence regarding effective mental health interventions for pregnant and parenting adolescents in low-resource African settings. Future research should therefore evaluate culturally appropriate interventions such as peer support programs, family strengthening approaches, economic empowerment interventions, school reintegration programs, and integrated maternal and paternal adolescent mental health services.

Further research should also pay greater attention to adolescent fathers, whose emotional experiences and support needs remain underexplored in many studies. Comparative studies between urban and rural settings, married and unmarried adolescents, and school-going versus out-of-school adolescents may also generate important contextual insights for targeted programming.

Acknowledgements

The authors acknowledge the adolescent mothers and fathers who shared their experiences and the health workers, caregivers, VHT members, and community leaders who participated in the study. Appreciation is also extended to the participating health facilities and communities in Mbarara District for their cooperation and support during data collection.

## Conclusions

This study provides important insights into the lived experiences, social support systems, and coping mechanisms among adolescent mothers and fathers with depression in southwestern Uganda. The findings show that parenting adolescents experience profound emotional, social, and economic challenges characterized by persistent worry, hopelessness, stigma, school dropout, poverty, partner abandonment, and social isolation. Depression among parenting adolescents emerged not simply as an individual psychological condition but as a reflection of broader structural inequalities, weak support systems, and community-level discrimination.

At the same time, the study found that family support, peer relationships, spirituality, and personal resilience play important protective roles in helping adolescents cope with early parenthood and emotional distress. However, these coping mechanisms were largely informal and often insufficient in the context of widespread poverty, limited mental health services, and persistent social stigma. The findings underscore the urgent need for comprehensive and adolescent-friendly interventions that integrate mental health support into maternal, reproductive, and community health services. Addressing the mental health needs of parenting adolescents requires coordinated responses that strengthen family and community support systems, reduce stigma, improve access to counseling and psychosocial care, support educational reintegration, and enhance economic opportunities for young parents.

## Appendices

Appendix I

In-depth interview guide for adolescent mothers and fathers (10-19) years with depression

Participant Information/Socio-Demographic Information

1.     How old are you? (age in full years)

2.     What is your gender? (male or female)

3.     What is your marital status? (single, co-habiting, I was dumped by the father of the child)

4.     What is your highest level of formal education? (mentions the class, or tertiary training)

5.     At what age did you first get pregnant or impregnated a girl? Can you share how you got your first pregnancy or first got a girl pregnant?

6.     How many children do you have now?

7.     Whom do you stay with? Do you live with your child(ren)?

8.     From where do you receive support for you and your child? What kind of support do you receive?

Lived Experiences of Depression as an Adolescent Parent

9.     Has getting pregnant at an early age affected you in any way? (please explain, e.g., in terms of education, family, mental health, societal beliefs)

10.  Can you describe your experience as an adolescent mother or father? (with feelings of joy, excitement, sadness, hopelessness, or anxiety)

11.  How do you perceive your mental health since becoming a parent at a young age? (mentally stable, depressed, anxious, hopeless)

12.  How has depression influenced your ability to parent and care for your child? (I love it, I don’t like it, I hate my child, I live in regret every day)

13.  Do you perceive any impact of depression on your future goals or aspirations?

Social Support Systems for Depressed Parenting Adolescents

14.  What strategies or coping mechanisms do you employ to manage your depressive symptoms?

15.  How do you manage challenges related to parenting while experiencing depression?

16.  Have you sought any form of professional help or mental health services for your depressive symptoms? (gone to health worker or health facility, counselor)

17.  What were your experiences like while seeking help due to depression in your community or from a health facility? (if respondent sought help from a health facility)

18.  What social support systems are available for you as a parenting adolescent in your community?

19.  What are the barriers preventing you from seeking or accessing these social support systems? (societal perceptions, shame, stigma, lack of services in the community)

20.  What suggestions do you have to help parenting adolescents cope with the situation they are in? (family level, community level, health facility, school, and national level)

I would like to thank you for sparing your time to share your experiences with us as an adolescent parent. Do you have any questions? 

Appendix II

Key informant interview guide for the social support systems (parents, local leaders, health workers, and VHT members)

Participant Information/Socio-Demographic Information

1.     How old are you?

2.     What is your gender?

3.     What is your marital status?

4.     What is your highest level of formal education?

5.     What is your position in this facility or community?

Parenting Adolescents' Lived Experiences

6.     How has getting pregnant at an early age affected adolescents in your community or institution? (please explain, e.g., in terms of education, family, mental health, societal beliefs)

7.     As a parent, local leader, health worker, can you describe the adolescents' experience as a young mother or father? (with feelings of joy, excitement, sadness, hopelessness, or anxiety)

8.     How do you perceive the adolescents' mental health since becoming parents at an early age? (mentally stable, depressed, anxious, hopeless)

9.     As a parent or health worker, or a local leader, have you witnessed any stigmatization or discrimination of these parenting adolescents due to their condition? Explain what happened.

Social Support Systems Available for Parenting Adolescents With Depression

10.  What support measures exist in your community or institution for depressed parenting adolescents?

11.  How do you manage challenges faced by parenting adolescents who are depressed?

12.  How do friends, family members, or community members contribute to these adolescents' mental health and well-being? (financial support, counseling)

13.  What can be done better to improve support given to depressed adolescent parents?

14.  What suggestions do you have for parenting adolescents to cope with the situation they are experiencing? (family level, community level, health facility, school, and national level)

I would like to thank you for sparing your time to share with us about depressed parenting adolescents. Do you have any questions? 
